# Non-targeted UHPLC-MS metabolomic data processing methods: a comparative investigation of normalisation, missing value imputation, transformation and scaling

**DOI:** 10.1007/s11306-016-1030-9

**Published:** 2016-04-15

**Authors:** Riccardo Di Guida, Jasper Engel, J. William Allwood, Ralf J. M. Weber, Martin R. Jones, Ulf Sommer, Mark R. Viant, Warwick B. Dunn

**Affiliations:** School of Biosciences, University of Birmingham, Edgbaston, Birmingham, B15 2TT UK; MRC-ARUK Centre for Musculoskeletal Ageing Research, University of Birmingham, Birmingham, B15 2TT UK; NERC Biomolecular Analysis Facility—Metabolomics Node (NBAF-B), University of Birmingham, Birmingham, B15 2TT UK; Phenome Centre Birmingham, University of Birmingham, Birmingham, B15 2TT UK; Institute of Metabolism and Systems Research, University of Birmingham, Edgbaston, Birmingham, B15 2TT UK

**Keywords:** UHPLC-MS, Metabolomics, Random forest, KNN, PQN normalisation, Glog transformation

## Abstract

**Introduction:**

The generic metabolomics data processing workflow is constructed with a serial set of processes including peak picking, quality assurance, normalisation, missing value imputation, transformation and scaling. The combination of these processes should present the experimental data in an appropriate structure so to identify the biological changes in a valid and robust manner.

**Objectives:**

Currently, different researchers apply different data processing methods and no assessment of the permutations applied to UHPLC-MS datasets has been published. Here we wish to define the most appropriate data processing workflow.

**Methods:**

We assess the influence of normalisation, missing value imputation, transformation and scaling methods on univariate and multivariate analysis of UHPLC-MS datasets acquired for different mammalian samples.

**Results:**

Our studies have shown that once data are filtered, missing values are not correlated with *m/z*, retention time or response. Following an exhaustive evaluation, we recommend PQN normalisation with no missing value imputation and no transformation or scaling for univariate analysis. For PCA we recommend applying PQN normalisation with Random Forest missing value imputation, glog transformation and no scaling method. For PLS-DA we recommend PQN normalisation, KNN as the missing value imputation method, generalised logarithm transformation and no scaling. These recommendations are based on searching for the biologically important metabolite features independent of their measured abundance.

**Conclusion:**

The appropriate choice of normalisation, missing value imputation, transformation and scaling methods differs depending on the data analysis method and the choice of method is essential to maximise the biological derivations from UHPLC-MS datasets.

**Electronic supplementary material:**

The online version of this article (doi:10.1007/s11306-016-1030-9) contains supplementary material, which is available to authorized users.

## Introduction

The application of Ultra High Performance Liquid Chromatography-Mass Spectrometry (UHPLC-MS) to acquire non-targeted metabolomics data is increasing in frequency. In 2014, there were 507 published papers in the 12 month period applying this instrumental platform as defined in PubMed compared to 12 and 179 in 2005 and 2010, respectively (search terms present in all fields = ‘metabolomics’ and ‘liquid chromatography’ and ‘mass spectrometry’). Following the acquisition of three-dimensional raw data (*m/z* vs. retention time vs. response), the first process to convert this raw data to biological knowledge is peak picking (or deconvolution) to align and integrate data across multiple samples. Software such as XCMS (Smith et al. 2006) and mzMine (Katajamaa et al. 2006) are freely available and commonly applied. The resulting data matrix is typically constructed with thousands of ‘metabolite features’ (m/z-retention time pairs) and tens-to-thousands of samples. Prior to univariate and multivariate data analysis this data matrix typically undergoes a number of processes including quality control (Dunn et al. 2011), missing value imputation, normalisation, scaling and transformation; here we will define this as ‘data processing’ and the processes applied can follow a specific workflow dependent on a number of factors including the structure of the data acquired and the subsequent data analysis techniques applied. A range of tools [e.g. MetaboAnalyst (http://www.metaboanalyst.ca/)], workflows [e.g. Galaxy-M (https://github.com/Viant-Metabolomics/Galaxy-M; Davidson et al. 2016) and Workflow4Metabolomics (Giacomoni et al. 2015)] and R packages [e.g. mixOmics; http://mixomics.qfab.org] are available to perform data processing. A random selection of 51 papers (10 % of all papers) published in this area in 2014 was investigated to define the different processing methods applied; SI1 lists the methods applied for normalisation, missing value imputation, data transformation and scaling. It is clearly evident that no single processing workflow is applied across the metabolomics community. Interestingly, a number of papers do not even define which processing methods were applied.

Variation in the measured response unrelated to the biological differences between samples can be observed in studies analysing tens-to-thousands of samples. These sources of variation include small changes in volume applied during sample preparation and sample injection and in instrument performance (changes in ionisation, ion transfer and detector efficiency). Normalisation is applied to correct for these unwanted peak intensity differences and to stabilise the variance within the dataset. Normalisation can be performed with or without applying an internal standard as a reference to calculate observed analytical errors. Normalization methods that are not based on internal standards often apply the sum, mean or the median of the responses of all metabolites across a sample as a normalization factor (Xia and Wishart 2011; Martucci et al. 2014; Kohl et al. 2012). However, some of these approaches (sum and mean) can introduce artificial correlations in the data in the case of large differences between the groups of samples in one or a few metabolites (Dieterle et al. 2006; Li et al. 2015). Probabilistic quotient normalisation (PQN) (Dieterle et al. 2006) was developed to reduce this effect and has successfully been applied for normalization in many metabolomics studies (Hrydziuszko and Viant 2012; Davies et al. 2014; Cottet et al. 2014). Different methods applying internal standards have been developed (De Livera et al. 2012). Other methods applying internal standards for UHPLC-MS (Sysi-Aho et al. 2007; Waybright et al. 2006) and GC–MS (Dunn et al. 2008b; Biais et al. 2009) for different biological samples have been investigated and reported.

Missing values in metabolomics datasets can be observed for three reasons: (1) metabolite is detected in one sample but is not present at any concentration in another sample; (2) metabolite is present in a sample but at a concentration less than the analytical method’s limit of detection, and (3) metabolite is present in a sample at a concentration greater than the analytical method’s limit of detection but the data processing software has not detected and reported the metabolite. Some software apply gap filling algorithms (Scheltema et al. 2011; Pluskal et al. 2010) though the majority of software do not apply these algorithms, including XCMS which is predominantly applied by the metabolomics community (in the period between April 2015 and March 2016 the package XCMS was downloaded 20,798 times from 6469 different IPs (https://bioconductor.org/packages/stats/bioc/xcms.html). Missing value imputation (MVI) is applied to logically replace missing values with a non-zero value while maintaining the data structure. This approach is primarily applied in multivariate analysis which typically operates most robustly with a dataset not containing missing values, though one example where missing values can be present is Bayesian PCA where missing value imputation is performed as part of the algorithm. Rubin identified three types of missing value occurrences (Rubin 1976): data can be missing completely at random (MCAR) when the missing-ness is unrelated to any observed variable or response, missing at random (MAR) when the missing-ness is related to one or more observed variables but not to the response, and missing not at random (MNAR) when the missing-ness is related to the response itself. Therefore the distribution of missing values can be random or systematic and in both cases the cause may be biological or technical (Little 1998; Hrydziuszko and Viant 2012). For example, if one class of samples does not contain a metabolite that is present in another class of samples, the missing values in the dataset are most probably occurring for a biological reason and can be defined as MNAR. However, if a metabolite which is present in the sample is not detected in the majority of or all samples then this is most probably a result of the metabolite concentration being lower than the analytical methods’ limit of detection; the missing values are a result of a combination of biological and technical issues and therefore the missing value can be accounted as MNAR. Another source of missing values caused by technical reasons are errors associated with peak picking software where the peak is present but the peak observed in the raw data is not reported, in this case the missing value can be accounted as MCAR.

A number of different missing value imputation (MVI) methods are available. These include small value replacement (SV) (Xia and Wishart 2011), mean replacement (MN) (Steuer et al. 2007), median replacement (MD) (Steuer et al. 2007), k-nearest neighbour (KNN) (Steuer et al. 2007; Troyanskaya et al. 2001), Bayesian PCA (BPCA) (Nyamundanda et al. 2010; Xia and Wishart 2011), multivariate imputation by chained equation MICE (van Buuren and Groothuis-Oudshoorn 2011) and Sangster’s method (Sangster et al. 2007). As the missing value imputation affects all of the following steps of the data processing and analysis pipeline it is extremely useful to identify the most appropriate method to apply in order to obtain the most robust results. Hrydziuszko and Viant (2012) have compared different commonly used missing value imputation methods for direct infusion Fourier transform ion cyclotron resonance mass spectrometry (DI FTICR-MS). Similarity between outcomes produced by different missing value imputation methods and imputation performance were evaluated and the results showed that KNN was the most robust missing value imputation method for DIMS metabolomics data. Gromski et al. (2014) published a study which explored the effects of different MVI methods on GC–MS datasets, evaluating their impact on classification performance in unsupervised and supervised multivariate models. That study concluded that Random Forest (RF) should be favoured as a MVI method. The application of MVI has also been investigated in transcriptomics and proteomics. Troyanskaya et al.(2001) examined the impact of missing values on statistical parameter evaluation for genomics, while the effect of the handling of missing values on univariate and multivariate statistics was studied by Scheel et al.(2005) for genomics and Pedreschi et al.(2008) for proteomics. While the first study recommended the use of an in-house package for MVI in transcriptomics, the second concluded that BPCA was the most efficient method for proteomics. A detailed investigation of how missing value imputation methods influence LC–MS metabolomics datasets and corresponding data analysis results has not been published to our knowledge.

Element-wise transformations of the data are carried out to correct for any data heteroscedasticity and any skewed distribution that is present. Transformation methods most frequently applied include logarithmic modifications [generalised logarithm (glog) or natural logarithm (nlog)] (Yau et al. 2014; Lopez-Sanchez et al. 2015; van der Kloet et al. 2013), often adding a constant value to the argument in order to cope with near-zero values (Mak et al. 2014). Scaling is performed to adjust for differences in fold change between metabolites which may be caused by large differences in the variation of the measured responses; however, the use of a scaling factor reduces such large differences to a relative value which is not dependant on the absolute abundance. A range of scaling methods has been applied in metabolomics including autoscaling (Jackson 1991), Pareto scaling (Eriksson et al. 1999), range scaling (Smilde et al. 2005) and VAST scaling (Keun et al. 2003). Different scaling and transformation methods have been assessed for GC–MS datasets (van den Berg et al. 2006) and a comparison between autoscaling and Pareto scaling has been performed for a single UHPLC-MS dataset (Masson et al. 2011). These studies concluded that autoscaling and range scaling were the most appropriate scaling method to apply in GC–MS metabolomics recommending these methods when metabolite abundance and fold change are not expected to influence the statistical multivariate model.

A number of papers investigating different data processing procedures have been published. Bijlsma et al. (2006) assessed different scaling, univariate analysis and multivariate analysis procedures in order to identify lipidomic biomarkers applying LC–MS. All data were normalised by reference to an internal standard as the only method assessed and only two scaling methods were tested (autoscaling and mean centering). The study produced a reproducible workflow for PLS-DA validation in order to detect low abundance biomarkers.

As detailed above a range of different data processing methods are applied but no systematic assessment of the integrated application of each of these methods has been published for UHPLC-MS metabolomics datasets. Here we assess different data processing methods, both singularly and combined, to define the data processing methods that are optimal for univariate (Mann–Whitney *U* test) and multivariate analysis (PCA, PLS-DA) methods and identify those data processing methods that are not appropriate for providing robust biological knowledge from UHPLC-MS data acquired for serum/plasma. We will define the impact of different data processing methods and formulate an appropriate ‘fit-for-purpose’ data processing workflow from these data. It must be noted that this approach does not deal with classification performance as studies dealing with the effect of different scaling procedures on classification performance has already been published (for example see Yang et al. 2015).

## Methods

### Data sets and raw data processing

#### Missing value imputation study

Four different non-targeted UHPLC-MS metabolomics datasets were employed to assess six different missing value imputation methods.

##### Datasets

1. Mouse serum from a study of ischemia following stroke acquired in negative ion centroid mode. The dataset consisted of 34 samples divided into five different classes and reported 4435 metabolite features. The *m/z* range was 100–1000 and the data were acquired applying a UHPLC Accela system coupled to an electrospray LTQ-Orbitrap Velos mass spectrometer (Thermo Scientific, UK) applying a method as previously described (Dunn et al. 2011).
2. Placental tissue extract from a study of normal and pre-eclamptic pregnancy as published previously (Dunn et al. 2012). Data for 24 samples were acquired in negative ion centroid mode with 3412 metabolite features. The *m/z* range was 100–1000 and data were acquired on a UHPLC-MS system (Waters Acquity UHPLC system and Thermo Scientific LTQ-Orbitrap XL).
3. Human urine samples; these data are currently not published. The dataset consisted of 48 samples acquired in positive ion profile mode with 3823 metabolite features. The *m/z* range was 100–1000 and data were acquired on a UHPLC-MS system (Thermo Scientific Dionex Ultimate 3000 UHPLC system and Thermo Scientific Q-Exactive).
4. Mammalian cellular extracts dataset; these data are currently not published. The dataset consisted of 88 fibroblast samples acquired in positive ion profile mode with 2008 metabolite features. The *m/z* range was 100–1000 and data were acquired on a UHPLC-MS system (Thermo Scientific Dionex Ultimate 3000 UHPLC system and Thermo Scientific Q-Exactive).

##### XCMS processing

The .RAW files produced were converted to .mzML format applying ProteoWizard 2.1 (Kessner et al. 2008) followed by deconvolution and peak alignment applying XCMS applying a previously described method (Dunn et al. 2008a).

##### Metabolite feature filtering

Firstly, missing value imputation was performed for each feature that was not detected in a single class but was detected in other classes. Applying R, the missing values in the single class were replaced by a value defined as the minimum peak area reported in the data matrix multipled by 0.5. Subsequently, features or samples containing more than 20 % missing values across all classes were deleted (peak filtering).

##### Missing Value Imputation methods

Normalisation by sum for each sample was applied (see Sect. 2.1.2.3). Five different missing value imputation methods were assessed:

1. Small value replacement (SV): for every metabolite feature the missing values were replaced by a value half of the minimum peak intensity of the entire dataset (Xia and Wishart 2011).
2. Mean replacement (MN): for every metabolite feature the missing values were replaced by the mean of the specific metabolite across all samples (Xia and Wishart 2011) (excluding the missing values in the calculation).
3. Median replacement (MD): for every metabolite feature the missing values were replaced by the median of the specific metabolite across all samples (Xia and Wishart 2011) (excluding the missing values in the calculation).
4. K-nearest neighbour imputation (KNN): the missing values are replaced by the average of the corresponding (feature specific) non-missing values in the k (here k = 10) closest features in terms of Euclidean distance of the responses across all the samples. Therefore a unique value is imputed for every missing value in a feature instead of using the same value multiple times as in approaches 1–3 (Xia and Wishart 2011; Hrydziuszko and Viant 2012).
5. Bayesian Principal Component Analysis replacement (BPCA): the missing values are replaced by the values obtained through principal component analysis regression with a Bayesian method. Therefore every imputed missing value does not occur multiple times neither across the samples nor across the metabolite features (Hrydziuszko and Viant 2012; Nyamundanda et al. 2010).
6. Random Forest imputation (RF): missing values are iteratively imputed using as a decisional criterion the proximity matrix generated by a RF classification computed across the total number of metabolites (Breiman 2001).

All the computations were performed using built-in R 3.0.2 functions except KNN which was performed using the package “impute”, BPCA which was performed using the package “pcaMethods”(Stacklies et al. 2007) and RF which was performed using the package “missForest”. “impute” and “pcaMethod” packages are freely available in Bioconductor (http://www.bioconductor.org/) while “missForest” is downloadable from the CRAN repository (https://cran.r-project.org/). Multivariate imputation by chained equation was not tested since it resulted in a computationally intense method while Sangster’s method was not performed due to lack of technical replicates, which is a common occurrence in non-targeted metabolomics studies.

##### Assessment of different MVI methods

In order to assess the performance of the different imputation methods each of the datasets described in Sect. 2.1.1.1 was treated as follows: only metabolite features (*m/z*-retention time pairs) with no missing values were retained; next a series of feature intensities in this data matrix were randomly selected, their intensities re-classified as ‘missing values’, until reaching a missing value frequency in the dataset of 10 %. Data were simulated as missing completely at random (MCAR) because the Pearson correlation coefficients did not show any relationship between *m/z*, response, retention time and frequency of missing values. Next, comparison of the original matrix (with no missing values) to the modified matrix (with randomly introduced missing values)—for each of the four datasets investigated—was performed applying normalised root mean squared error (NRMSE) for every imputation method. The root mean squared error was calculated on the difference between original and imputed values and normalised by the mean value of the matrix.

##### Calculation of Pearson correlation coefficients

For each metabolite feature the *m/z*, retention time, mean response and number of missing values were calculated. Pearson correlation coefficients were calculated for (i) *m/z* vs. number of missing values; (ii) retention time vs. number of missing values, and (iii) mean response vs. number of missing values.

#### Data processing study

##### Dataset

A single UHPLC-MS dataset was used to assess the effect of different combinations of processing methods in relation to response and fold change. Human serum was acquired in positive ion mode for 64 samples (46 biological samples and 18 QC samples). The total number of detected metabolite features was 3837. The *m/z* range applied was 100-1000 and the data were acquired on a Ultimate3000 UHPLC system coupled to a LTQ-FT Ultra mass spectrometer (Thermo Scientific, UK). Data were processed applying XCMS as defined in Sect. 2.1.1.2.

##### Construction of modified dataset

We artificially modified peak intensities to introduce known metabolic differences between groups and this provided us with a target for discovering these artificial peak intensities using both univariate and multivariate statistics. The effects of the processing steps on our ability to re-discover these known metabolic differences were evaluated. The 46 biological samples were randomised into two classes with the median fold change between class A and class B in the range 0.8–1.2. Mann–Whitney *U* test defined no metabolite features that were statistically significant (p < 0.05). All metabolite features were separated into three blocks based on response (the mean response was calculated across all features, the vector of the means was then ordered and split into three sections with each section defined as 0–33 % (low), 34–66 % (medium) and 67–100 % (high) of the range of means). Thirty-two metabolite features in class A were randomly chosen (applying sample() random function in R) from each of the three blocks and multiplied by a factor between 0.1 and 10 (0.1 to 2.0 in steps of 0.1 and from 2.5 to 10.0 in steps of 0.5). This modified dataset was applied for comparison of different processing methods. A flow chart is available in SI2.

##### Normalisation methods

The modified dataset was normalised by sum or PQN.

- Normalisation by sum: each value in a row (sample) is divided by the total sum of the row (sample) and multiplied by 100; the unit is %.
- PQN: for every feature the mean response is calculated across all QC samples. A reference vector is then generated. The median between the reference vector and every sample is computed obtaining a vector of coefficients related to each sample. Each sample is then divided by the median value of the vector of coefficients; this median value is different for each sample. This method was adapted by Dieterle et al. (2006). Its purpose is to take into account the concentration changes of some metabolite features that affect limited regions of the data.

Other normalisation methods requiring internal standards or technical replicates were not investigated because the analytical samples contained no internal standard and technical replicates were not acquired.

##### Missing Value Imputation methods

Six missing value imputation methods were assessed as defined in Sect. 2.1.1.4.

##### Transformation methods

Three different transformation methods (van den Berg et al. 2006) were assessed:

- Generalised logarithm (glog) (Parsons et al. 2007): every value is transformed according to the equation
  $$z = \ln \left( {y + \sqrt {y^{2} + \lambda } } \right)$$
  where y is the untransformed value, z is the transformed value and λ is a parameter which is iteratively computed (from a series of technical replicates, in this case QC samples) in order to minimise the variation;
- Natural logarithm (nlog): every value is transformed in the corresponding natural logarithm;
- Inverse hyperbolic sine (IHS)(Mak et al. 2014): every value is transformed according to the equation:
  $$z = \left( {\ln (y + \sqrt {y^{2} + 1)} } \right)$$

##### Scaling methods

Four different (peak-wise) scaling approaches were assessed:

- Autoscaling: every peak is mean centered and divided by the standard deviation of the column. This treatment makes the standard deviation of each metabolite equal to 1. Autoscaling, along with range scaling, is not affected by the feature abundance (Jackson 1991).
- Pareto scaling: every peak is mean centered and divided by the square root of the standard deviation of the column (Eriksson et al. 1999). The influence of noise variables on the multivariate model is reduced compared to autoscaling.
- Range scaling: every peak is mean centered and divided by the numerical difference between the maximum and minimum values of the column (Smilde et al. 2005).
- Vast scaling: every peak is autoscaled and divided by the coefficient of variation. It is particularly suited for metabolites bearing small fold changes (Keun et al. 2003).

All processing was carried out using built-in R 3.0.2 functions. The source code for many of the steps applied can be found in the Galaxy-M repository https://github.com/Viant-Metabolomics/Galaxy-M. All possible permutations of normalisation, missing values imputation, transformation and scaling (see SI3) were explored and applied on the modified dataset described.

##### Univariate and multivariate data analysis

The Shapiro–Wilk test of normality was applied for all metabolite features to assess whether the data were normally distributed (the null hypothesis is that the distribution does not differ from a normal distribution; p < 0.05 defines that the distribution is not normal). This was performed on data before any normalisation, MVI, scaling and transformation and after normalisation, MVI, scaling and transformation.

Univariate analysis was performed applying the Mann–Whitney *U* test and Students *t* test (non-parametric and parametric, respectively) between class A and class B. Data for QC samples were removed from the datasets prior to univariate analysis. The Benjamini–Hochberg (Benjamini and Hochberg 1995) false discovery correction for multiple comparisons was applied afterwards.

Multivariate analysis was performed applying Principal Component Analysis (PCA) and Partial Least Squares-Discriminant Analysis (PLS-DA), QC samples were excluded from the analysis prior to PLS-DA analysis. The R packages mixOmics and pcaMethods were used for this purpose. Further statistical analysis consisted of t-test or Mann-Whitney U-test performed on the PCA scores in order to identify statistically significant clustering patterns. Prior to these procedures a Shapiro–Wilk test was carried out to assess the normality of the score matrix in order to apply parametric or non-parametric statistics. For PLS-DA, the classification performance was identified through the application of cross-validation and calculation of R^2^ and Q^2^ values. The optimal number of components was evaluated applying a tenfold cross validation.

*Analysis of the univariate outcome* The Mann–Whitney U-test and t-test between class A and B were calculated for each combination of normalisation, missing value imputation, transformation and scaling methods (a total of 280 permutations). The total number of significant peaks (q < 0.05) and the number of the 96 metabolite features (with deliberately modified intensities) showing statistical significance were both reported. The number of false positive statistically significant metabolite features was calculated by subtracting the number of intensity-modified significant peaks from the total number of significant peaks. Here, by “false positive”, we indicate peaks that were incorrectly marked as significantly different; the intensities of these peaks were not altered, hence bearing a fold change between classes in the range 0.8 and 1.2 and not being statistically significant in the original dataset.

*Analysis of the multivariate outcome* Following PCA and PLS-DA analysis all the metabolite features that were intensity-modified were ranked according to their absolute loading value on PC1, PC2 and a combination of PC1 and PC2, and on latent variable 1, latent variable 2 and a combination of both for PLS-DA. The range of ranks was reported for the top ten highest ranked metabolite features.

The permutations (the 280 different combinations of normalisation, MVI, scaling and transformation) were sorted including the entries having a fold change effect and lacking an abundance effect in PC1 and PC2. These entries were sorted according to the p-value (low to high) produced by the PC1 score plot separation between classes and according to the % variance contribution for PC1 + PC2 as a second level. For PLS-DA the permutations were sorted by R^2^ value (high to low) and differences between R^2^ and Q^2^ of less than 0.20 (low to high).

## Results and discussion

### Missing value imputation study

The percentage of missing values was calculated and an assessment to determine whether missing values were correlated with *m/z*, retention time or response was performed for four different datasets (mouse serum, human urine, placental tissue, mammalian cell extract) acquired applying two different analytical methods (reversed phase and HILIC) and three different UHPLC-MS platforms (Accela UHPLC coupled to LTQ-Orbitrap Velos, Ultimate3000 coupled to LTQ-FT Ultra, and Ultimate3000 coupled to Q Exactive). Table 1 defines the percentage missing values and Pearson correlation coefficients for each dataset. In this paper we define a metabolite feature as a *m/z*-retention time pair with a single metabolite typically being detected as more than one metabolite feature.

**Table 1:** Summary of the percentage of missing values present in four datasets and the correlation of missing values observed with *m/z*, retention time and response

Dataset	Mouse serum	Placental tissue	Human urine	Mammalian cell extract
Metabolite features before filtering	4435	3412	3823	2008
Missing values before filtering (%)	15.0	10.2	14.0	8.7
Metabolite features after filtering	2996	2622	2684	1598
Missing values after filtering (%)	4.5	2.8	5.0	3.7
Pearson coefficient (missing values vs. mean abundance)	−0.05	−0.12	−0.05	−0.08
Pearson coefficient (missing values vs. *m/z* values)	0.07	−0.02	0.30	0.35
Pearson coefficient (missing values vs. retention time)	0.02	−0.11	0.03	−0.07

Filtering was performed as defined in Sect. 2.1.1.3

The percentage of missing values ranged from 8.7 to 15.0 % before filtering and 2.8 to 5.0 % after filtering. A relatively strict filter was applied, where any metabolite feature with >20 % missing values was removed from the dataset. Correlations between *m/z* and the number of missing values, retention time and the number of missing values, and intensity and the number of missing values were close to zero. These results show that the number of missing values is not correlated with *m/z*, retention time or response across the complete data for each dataset. The structure of missing values in relation to *m/z*, retention time or response is shown in SI4. For two of four datasets (mammalian cell extract and placental tissue) some structure is observed when investigating missing values and retention time. Here a lack of missing values is observed around 150–250 s, though the number of metabolite features detected in this retention time range is lower than in any other 100 s range. We therefore assume that missing values occur at random across the dataset and are primarily a result of peak picking software not reporting metabolite peaks; i.e. the peaks are missing because of the genuine absence of metabolites or because they are present but at a concentration lower than the analytical method’s limit of detection.

To assess six different MVI methods (SV, MN, MD, KNN, BPCA and RF) the four datasets discussed above were filtered to remove metabolite features which contained one or more missing value. Missing values were then randomly introduced into each of the datasets to a frequency of 10 %. This frequency of 10 % was chosen as it was equivalent to the highest missing value frequency in six further datasets collected with different instrument manufacturers (4 datasets collected on Agilent Q-TOF systems and 2 datasets collected on Waters Q-TOF systems) which presented a distribution of missing values after peak filtering of 2.3–10.5 %. These datasets are detailed in SI5. The same range for the four datasets discussed above was 2.8–5.0 %. The similarity between the original matrix with no missing values and the modified matrix containing imputed missing values was calculated applying normalised root mean squared error (NRMSE) for each of the six missing value imputation methods. The results are shown in Table 2.

**Table 2:** Normalised root mean squared errors (NRMSE) for four datasets for comparison of six different missing value imputation methods

MVI method	Mouse serum	Placental tissue	Human urine	Mammalian cell extract
Small value replacement	9.99	3.64	7.66	5.53
Mean	1.82	0.66	1.47	1.01
Median	1.60	0.68	1.49	1.01
K-nearest neighbours	1.29	0.58	1.44	0.54
Bayesian principal components analysis	1.30	0.62	1.49	1.12
Random forest	0.75	0.45	1.16	0.37

An NRMSE close to zero implies the imputation algorithm has most correctly predicted the missing values

The results show that small value replacement, with the largest NRMSE values, is the least optimal method for missing value imputation. This is expected as small value imputation would be expected to work when missing values are related to low responses; however Pearson correlation analysis showed no correlation between response and missing values. RF achieved the lowest NRMSE values in all four datasets with marked improvement compared to all the other imputation methods. KNN and BPCA also performed quite well with KNN slightly out performing BPCA for placental tissue and mammalian cell extracts datasets while BPCA performing similarly to KNN in mouse serum and human urine datasets. Overall, RF seems to be the best imputation method in all cases tested. The major drawback of RF is the computational time which was typically greater than 15 min in the study reported here. KNN and BPCA, despite achieving a worse performance compared to RF require considerably faster computation times. The use of MN and MD showed comparable results for placental tissue and mammalian cell extract datasets while they tended to be less efficient in the mouse serum and human urine datasets. Conclusively, it is recommended that RF is applied for missing value imputation for multivariate analysis, consistent with the finding for GC–MS metabolomics data (Gromski et al. 2014). We do not recommend the use of MVI for univariate analysis because of the potential to change the distribution within each class. Even though RF provided the smallest NRMSE, the values were not zero and therefore indicative of a ‘perfect’ missing value algorithm.

### Data processing study

A sample set containing 46 biological samples analysed in positive ion mode applying UHPLC–MS was randomised into two classes containing 22 (class A) and 24 samples (class B); the R function applied does not provide the same number of samples in each class. Univariate and multivariate analysis was performed prior to any dataset modification. The Mann–Whitney U-test did not identify any statistically significant peaks (p < 0.05) and PCA reported the samples as randomly scattered across the scores plot for PC1-3 (data not shown). PLS-DA did not report a cross-validated model. These results show that no separation of classes A and B was observed before the dataset was modified (data not shown).

After all metabolite features were ranked according to response and grouped into three classes (low, medium and high response), 32 metabolite features in each class were randomly chosen. For each class the metabolite features were randomised into a rank order and then the response was multiplied by a factor between 0.1 and 10. Following this process there were 7.8 % of all reported values which were defined as missing values and 65.3 % of features were shown to have at least one sample with a missing value. The normality of the resulting dataset was assessed applying the Shapiro–Wilk test showing a high percentage of features not following a normal distribution before and after log transformation as shown in SI6.

Consequently the modified dataset was analysed applying non-parametric univariate (Mann–Whitney *U* test) as well as multivariate (PCA, PLS-DA) methods following different permutations of normalisation, missing value imputation, transformation and scaling methods being applied. Two normalisation (PQN and SUM), seven missing value imputation (none, SV, MN, MD, KNN, BPCA and RF), four transformation (none, glog, ihs, nlog) and five scaling (none, autoscaling, Pareto scaling, range scaling and Vast scaling) methods were assessed. 280 different permutations were assessed as shown in SI3.

#### Univariate analysis

The results for all the permutations are shown in SI7 and a summary presented in Table 3, where each unique set of results is shown and where one row can represent multiple permutations; for example, ‘all’ defines that all of the methods applied produced the same result. The results show that no permutation of data processing methods is ideal, as no method led to 96 true positive (statistically significant) features and zero false positives. Three permutations provided 81 true positive and zero false positive results. Two of these applied PQN normalisation and one SUM normalisation. Interestingly, two permutations applied RF missing value imputations and one did not apply any missing value imputation. Although RF missing value imputation provided the best results in this comparison we still recommend that RF is not applied for univariate analysis. This is because the NRMSE values reported in 3.1 were not zero (indicative of a ‘perfect’ missing value algorithm) and therefore the data structure is still altered.

**Table 3:** Summary of the number of the 96 modified metabolite features defined as statistically significant (q < 0.05) and the number of metabolite features falsely reported as statistically significant (q < 0.05) for all of the different data processing methods applied

Normalisation	Missing value imputation	Transformation	Scaling	True positive results	False positive results
PQN/SUM	RF	All	All	81	0
PQN	None	All	All	81	0
PQN	MN/MD	All	All	80	0
SUM	None	All	All	80	0
SUM	MN/MD/SV	All	All	79	0
PQN	SV	All	All	77	0
SUM	BPCA	glog	Range/Autoscaling/VAST	81	1
SUM	KNN	All	All	81	3
SUM	BPCA	All	None/Pareto	81	3
PQN	KNN	All	All	82	5
PQN	BPCA	All	All	82	6

All defines that all methods provided the same result. The closer the number of significant modified features to 96 implies the data processing has performed more ideally

*PQN* probabilistic quotient normalization, *RF* random forest, *MN* mean, *MD* median; *SV* small value, *KNN* k-nearest neighbour, *BPCA* Bayesian principal components analysis, *glog* generalised log

The normalisation method had a minimal effect, with PQN and SUM reporting 81 and 80 statistically significant metabolites. KNN and BPCA missing value imputation methods and all scaling methods resulted in false positives for both normalisation processes and are not fully appropriate to apply for univariate analysis. Sixteen and fifteen metabolites were not reported as statistically significant for the SUM and PQN normalisation methods. The metabolites reported as not statistically significant were primarily those with a fold change in the range 0.8–1.2 for all three response classes (low, median, high). This shows that the inter-subject variation in the dataset is greater than the variation associated with the modifications performed for these 15 metabolite features. At fold changes less than 0.8 and greater than 1.2 there was no significant effect of fold change or response on the p-values reported for any metabolite within a single permutation. Our results show that the use of SUM or PQN normalisation with no MVI and no scaling or transformation should be applied for univariate analysis. It should be noted that SUM normalisation cannot deal well with large differences in a few metabolites in large datasets and so PQN normalisation is recommended (Kohl et al. 2012). This conclusion should not be interpreted as a rigid rule, instead it offers guidance to the user. Indeed the treatment performed for univariate analysis is heavily affected by the purpose of the study. The results we report here are somewhat consistent with the conclusions reported by Hrydziuszko et al.^4^ for DIMS data, though this reported study did not assess RF missing value imputation.

##### Students *t* test

A similar investigation was performed as for Sect. 3.2.1 with the single difference being the use of the parametric students *t* test. Many research groups apply parametric statistical tests and there is a general assumption that log transformations will convert data into a normal distribution, which we investigated here. As defined in SI6, the percentage of metabolite features not demonstrating a normal distribution with no glog transformation ranged from 39.6 to 41.6 % with glog transformation reducing the number of metabolite features showing a non-normal distribution by less than 12 %. In conclusion, although log transformations increase the number of features which are normally distributed, the percentage of features which are not normally distributed is still high (greater than 50 %). To evaluate the use of the Students *t* test and Mann–Whitney *U* test we applied the Students t-test and compared the results to those obtained for the Mann–Whitney *U* test (see Sect. 3.2.1). The data applying Students *t* test are shown in SI8. The manipulated features which were not statistically significant were again the ones multiplied by a factor ranging between 0.8 and 1.2 apart from a few exceptions. As a general conclusion, the same number of true positives and false positives were reported when applying both the parametric and non-parametric statistical methods.

#### Multivariate analysis

##### PCA

**Fig. 1:**
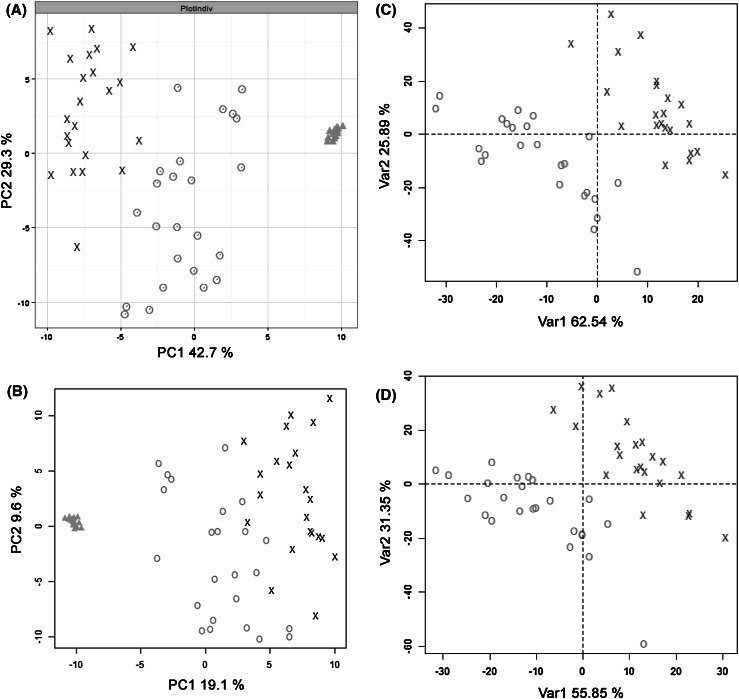
Examples of PCA and PLS-DA scores plots for acceptable and not acceptable data processing methods **a** PCA scores plot for data processed applying RF missing value imputation, SUM normalisation, glog transformation and no scaling which is defined as an acceptable method; 100 % variance accounted for in PC1 and 2, PC1 p = 4.8E^−12^; **b** PCA scores plot for data processed applying small value missing value imputation, SUM normalisation, glog transformation and no scaling which is defined as not an acceptable method; 25.7 % variance accounted for in PC1 and 2, PC1 p = 1.8E^−7^; **c** PLS-DA scores plot for data processed applying KNN missing value imputation, PQN normalisation, glog transformation and no scaling which is defined as an acceptable method; R^2^ = 0.61, Q^2^ = 0.46; **d** PLS-DA scores plot for data processed applying small value missing value imputation, SUM normalisation, glog transformation and no scaling which is defined as not an acceptable method; R^2^ = 0.42, Q^2^ = 0.31. *Red* circles = Class A; *black* crosses = Class B; *Green* triangles = QC sample (Color figure online)

The dataset was applied to assess all 280 permutations of normalisation, missing value imputation, transformation and scaling followed by PCA analysis. The purpose of this study was to identify data processing methods driven by fold change but not abundance (as is applied in many but not all metabolomics studies). Indeed such an outcome is desirable since it is rather common for metabolomics datasets to present the majority of detected metabolites with relatively low abundance. Here for example, 2843 features out of 3837 were present in the 25th quartile thus highlighting the potential importance of low abundance peaks. The results are shown in SI9 and the top 10 permutations showing (1) separations related to fold change only; (2) the highest variance observed in PC1 + PC2 and (3) the most statistically significant differences in PC1 are shown in Table 4. The first ten ranked permutations all applied RF for missing value imputation and either SUM or PQN normalisation. The first five ranked permutations showing the highest statistical significance all applied no scaling and applied glog, nlog or IHS transformations. SUM normalisation, RF missing value imputation, glog transformation and no scaling contributed 100 % variance in PC1 + PC2 and the most statistically significant p-value for PC1. While the combination of SUM or PQN normalisation and no missing value imputation may be addressed as a solution, it must be remarked that the PCA computation on datasets including missing values is slower compared to the calculation on imputed datasets because of the use of the NIPALS algorithm which imputes missing values; furthermore the evaluation of PLS-DA models from datasets containing missing values often encounters several technical issues (e.g. cross-validation methods do not work well when missing values are present). PQN, despite achieving lower p-values compared to SUM permutations, still produces good separation across both principal components. As discussed above, SUM normalisation cannot deal well with large differences in a few metabolites in large datasets and so PQN normalisation is recommended (Kohl et al. 2012). It is notable that once a transformation is performed scaling is not necessary to obtain PCA models that show the most statistically significant p-value for PC1. Examples of PCA scores plots for a method which is appropriate and is not appropriate is shown in Fig. 1.

**Table 4:** Summary of the top ten permutations according to p-value achieved for PC1 scores values

Normalisation	MVI	Transformation	Scaling	Variance (PC1; %)	Variance (PC2; %)	P-value (PC1)
SUM	RF	glog	None	42.7	39.3	4.82E−12
SUM	RF	nlog	None	44.7	28.6	1.66E−08
PQN	RF	glog	None	43.9	28.7	1.66E−08
PQN	RF	IHS	None	44.7	28.6	1.83E−07
PQN	RF	nlog	None	44.7	28.6	1.83E−07
SUM	RF	glog	Pareto	41.5	31.2	0.01601
SUM	RF	nlog	Pareto	43.2	30.4	0.02768
PQN	RF	nlog	Pareto	42.1	31.2	0.02934
PQN	RF	IHS	Pareto	42.1	31.2	0.02934
PQN	RF	glog	Pareto	41.7	31.5	0.03482

The greater the combined percentage variance for PC1 and PC2 and the lowest p-values for PC1 and PC2 implies the data processing has performed more ideally

*PQN* probabilistic quotient normalization, *RF* random forest, *glog* generalised log, *nlog* normal log, *IHS* inverse hyperbolic sine

##### PLS-DA

The dataset was applied to assess all 280 permutations of normalisation, missing value imputation, transformation and scaling followed by PLS-DA analysis. The results are shown in SI10 and the top eight ranked permutations based on the highest R^2^ value with a R^2^ − Q^2^ difference of less than 0.20 are shown in Table 5. The permutations where higher R^2^ values were observed show no distinct trend. There is no significant advantage obtained by applying SUM normalisation or PQN normalisation. KNN and BPCA missing value imputation operate more effectively than other imputation methods, including RF. The highest ranked permutations with the smallest R^2^ − Q^2^ difference were (1) PQN normalisation, BPCA MVI, glog transformation and range scaling and (2) SUM or PQN normalisation, KNN MVI, glog transformation and no scaling. Importantly, the optimal data processing methods for PLS-DA are different to the optimal methods for PCA. Examples of PLS-DA scores plots for a method which is appropriate and is not appropriate is shown in Fig. 1.

**Table 5:** Summary of the top 8 data processing methods according to the PLS-DA R^2^ values

Normalisation	MVI	Transformation	Scaling	R^2^	Q^2^	R^2^ − Q^2^
PQN	BPCA	nlog	Pareto	0.63	0.47	0.16
PQN	KNN	glog	None	0.61	0.46	0.15
PQN	BPCA	nlog	None	0.59	0.44	0.15
SUM	KNN	glog	None	0.59	0.51	0.08
SUM	BPCA	nlog	None	0.57	0.40	0.16
PQN	BPCA	IHS	None	0.56	0.38	0.18
PQN	BPCA	glog	rg	0.56	0.55	0.01
SUM	KNN	nlog	Auto	0.55	0.40	0.16

*PQN* probabilistic quotient normalization, *KNN* k-nearest neighbour, *BPCA* Bayesian principal components analysis, *glog* generalised log, *nlog* normal log, *IHS* inverse hyperbolic sine, *rg* range scaling, *Auto* autoscaling

### Data processing workflow

Applying the conclusions constructed from the data reported in this paper we have constructed a standardised data processing workflow for all mammalian sample datasets we study. This workflow is described in SI11 and includes peak picking, quality control, metabolite annotation, metabolite feature filtering, missing value imputation, normalisation and transformation processes applicable for both univariate and multivariate (PCA and PLS-DA) data analysis methods.

## Concluding remarks

This study has highlighted important relationships between normalisation, missing value imputation, transformation and scaling methods and how these should be applied prior to univariate and multivariate analysis. Once data is filtered, missing values are not correlated with *m/z*, retention time or response; instead missing values are randomly observed in datasets and are potentially a cause of errors in peak picking software. As has been reported previously, many missing value imputation methods negatively influence univariate analysis outcomes though we have shown here that the RF missing value imputation method performs equivalently to no missing value imputation. We recommend that no missing value imputation, no scaling and no transformations are used prior to univariate statistical analysis. When using SUM or PQN normalisation with RF missing value imputation or no missing value imputation and no other data processing methods applied, a high number of metabolite features were determined to be statistically significant while the false positive rate was 0 %. Normalisation, missing value imputation, scaling and transformation all impacted on the results observed for PCA and PLS-DA; datasets treated differently resulted in diverse clustering trends. It has been found for PCA that SUM or PQN normalisation, in combination with RF missing value imputation, glog transformation, and no scaling highlights the metabolite features with a significant fold change between classes regardless of the metabolite feature response with the highest percentage variance explained in PC1 and PC2 and with the most statistically significant p-value for PC1. We recommend this combination of data processing methods for PCA including the use of PQN rather than SUM normalisation because SUM normalisation has been shown to not be robust when a small number of metabolites with a large fold change is present. RF was reported as being the most valid missing value imputation method for PCA. For PLS-DA, KNN and BPCA missing value imputation operate more effectively than other imputation methods, including RF. The highest ranked permutations with the smallest R^2^ − Q^2^ difference were (1) PQN normalisation, BPCA MVI, glog transformation and range scaling and (2) SUM normalisation, KNN MVI, glog transformation and no scaling. We recommend the second of these permutations for PLS-DA analysis. Therefore we conclude that the best data processing procedures to apply when performing UHPLC–MS driven non-targeted metabolomics are different for univariate, PCA and PLS-DA methods when searching for the biologically important metabolite features independent of response. Importantly, our evaluation is based on classification and the use of Pareto scaling is recommended when determining the metabolites of biological significance in multivariate analysis.

## Electronic supplementary material

Below is the link to the electronic supplementary material.

Supplementary material 1 (XLSX 50 kb)

Supplementary material 2 (PDF 436 kb)

Supplementary material 3 (PDF 131 kb)

Supplementary material 4 (PDF 32 kb)

Supplementary material 5 (XLSX 14 kb)

Supplementary material 6 (PDF 237 kb)

Supplementary material 7 (XLSX 10 kb)

Supplementary material 8 (PDF 257 kb)

Supplementary material 9 (XLS 59 kb)

Supplementary material 10 (XLS 132 kb)

Supplementary material 11 (XLSX 54 kb)
