# Supplementary material for: Non-targeted UHPLC-MS metabolomic data processing methods: a comparative investigation of normalisation, missing value imputation, transformation and scaling
Source: Metabolomics. 2016 Apr 15;12:93. doi: 10.1007/s11306-016-1030-9 (PMC4831991; doi:10.1007/s11306-016-1030-9)
Supplement: Supplementary file 2 — Supplementary material 2 (PDF 436 kb) [file 11306_2016_1030_MOESM2_ESM.pdf]

SI 1. Summary of the percentage of missing values present in four datasets and the correlation of missing values observed with *m/z*, retention time and response. Filtering was performed as defined in section 2.1.1.3.

**Processing method**	**Methods applied (number of papers reported)**
Normalisation	PQN (2), normalization to IS (9), SUM (3), mean/median normalisation (3), other (3), not reported (31)
Missing value imputation	KNN (1), mean replacement (1), small value replacement (3), not reported (46)
Transformation	Natural logarithm (12), Log_2(5), Log_{10}(1), other (1), not reported (32)
Scaling	Autoscaling (11), Pareto scaling (6), level scaling (1), not reported (33)
