# Supplementary figures and images for: Non-targeted UHPLC-MS metabolomic data processing methods: a comparative investigation of normalisation, missing value imputation, transformation and scaling
Source: Metabolomics. 2016 Apr 15;12:93. doi: 10.1007/s11306-016-1030-9 (PMC4831991; doi:10.1007/s11306-016-1030-9)

**SI 11.** Data processing workflow applied for untargeted UHPLC-MS studies

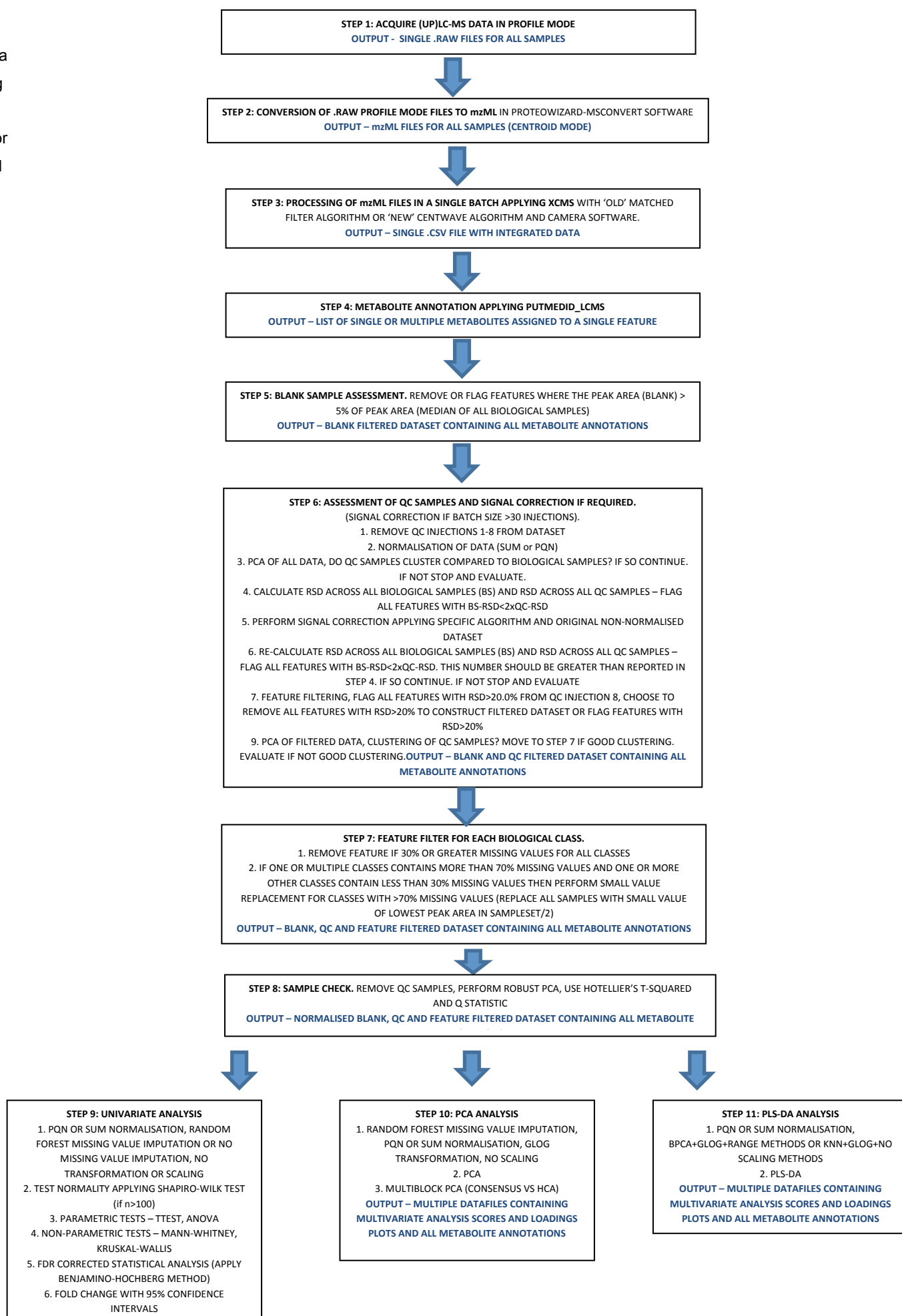

Supplement: Supplementary file 3 — Supplementary material 3 (PDF 131 kb) [file 11306_2016_1030_MOESM3_ESM.pdf]
