# Supplementary material for: Non-targeted UHPLC-MS metabolomic data processing methods: a comparative investigation of normalisation, missing value imputation, transformation and scaling
Source: Metabolomics. 2016 Apr 15;12:93. doi: 10.1007/s11306-016-1030-9 (PMC4831991; doi:10.1007/s11306-016-1030-9)
Supplement: Supplementary file 4 — Supplementary material 4 (PDF 32 kb) [file 11306_2016_1030_MOESM4_ESM.pdf]

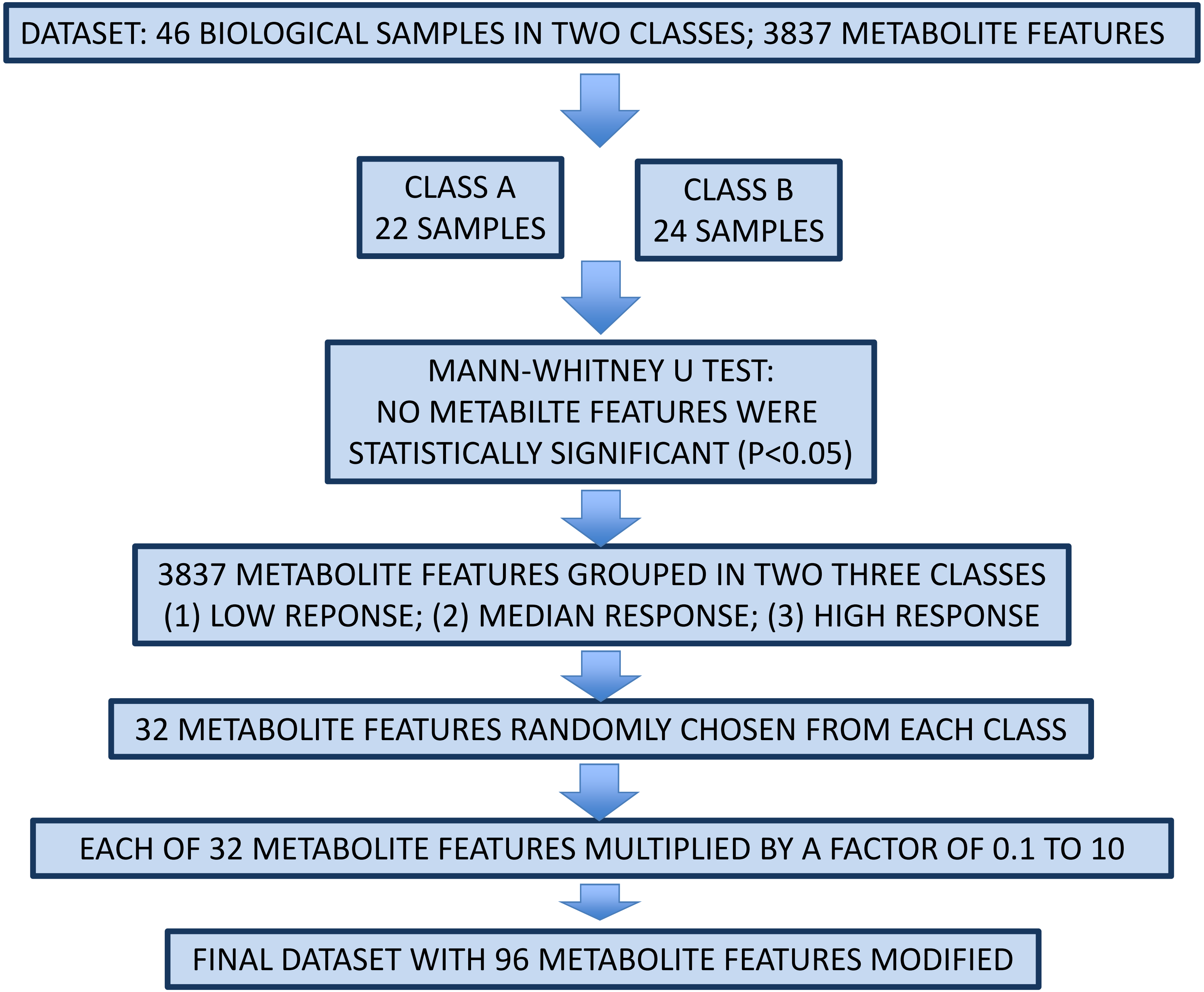
DATASET: 46 BIOLOGICAL SAMPLES IN TWO CLASSES; 3837 METABOLITE FEATURES
CLASS A
22 SAMPLES
CLASS B
24 SAMPLES
MANN-WHITNEY U TEST:
NO METABILTE FEATURES WERE
STATISTICALLY SIGNIFICANT (P<0.05)
3837 METABOLITE FEATURES GROUPED IN TWO THREE CLASSES
(1) LOW REPONSE; (2) MEDIAN RESPONSE; (3) HIGH RESPONSE
32 METABOLITE FEATURES RANDOMLY CHOSEN FROM EACH CLASS
EACH OF 32 METABOLITE FEATURES MULTIPLIED BY A FACTOR OF 0.1 TO 10
FINAL DATASET WITH 96 METABOLITE FEATURES MODIFIED
