# Supplementary material for: Non-targeted UHPLC-MS metabolomic data processing methods: a comparative investigation of normalisation, missing value imputation, transformation and scaling
Source: Metabolomics. 2016 Apr 15;12:93. doi: 10.1007/s11306-016-1030-9 (PMC4831991; doi:10.1007/s11306-016-1030-9)

SI4: Plots describing the distribution of missing values in relation to m/z, retention time (RT) and response for four different datasets (human cell line, mouse serum, human placental tissue and human urine);

Human cell line

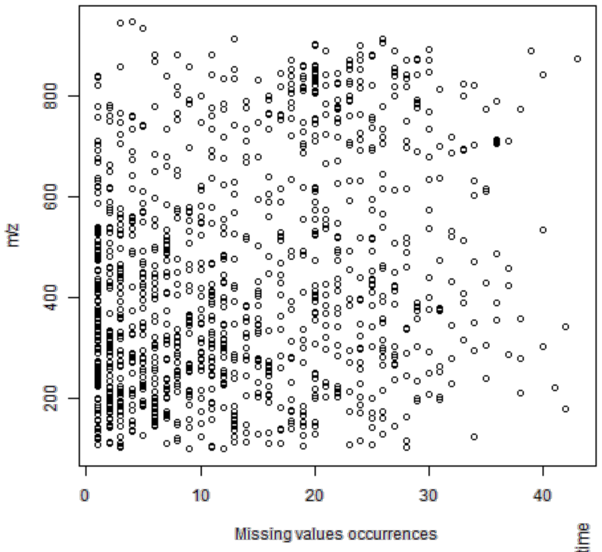

m/z

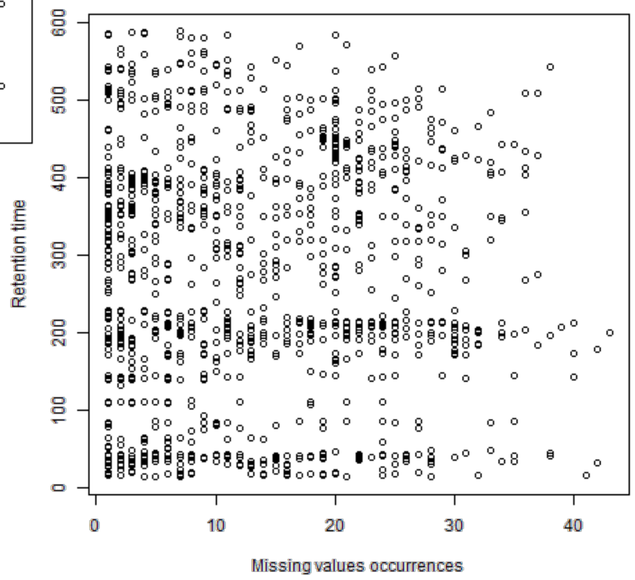

Retention time

Response

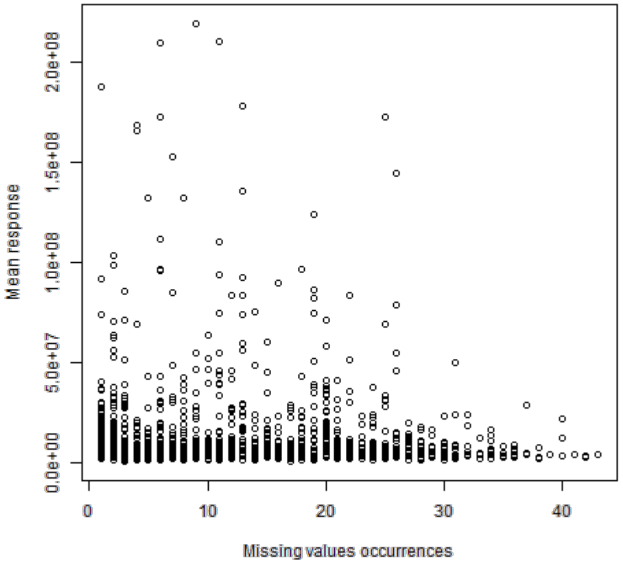

Mouse serum

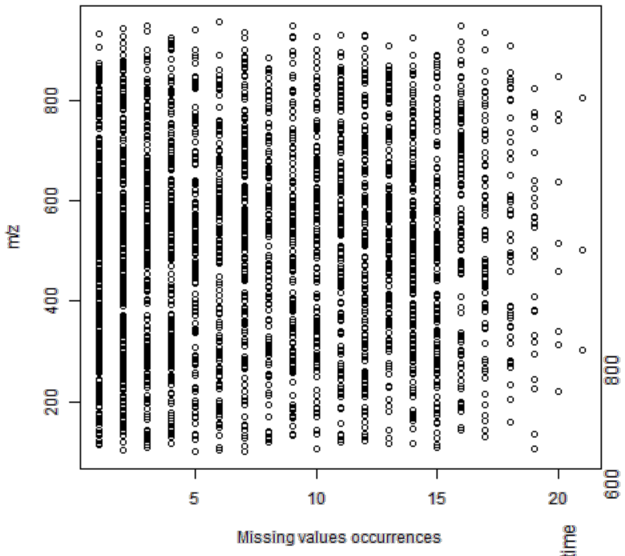

m/z

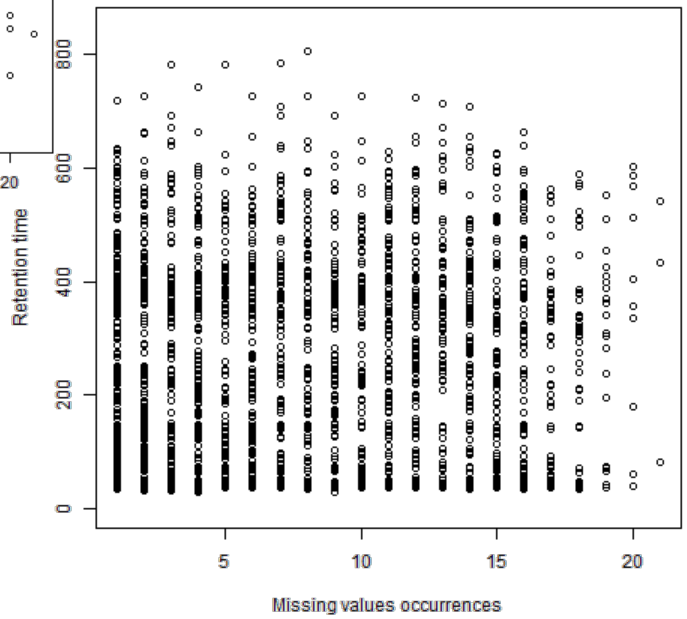

Retention time

Response

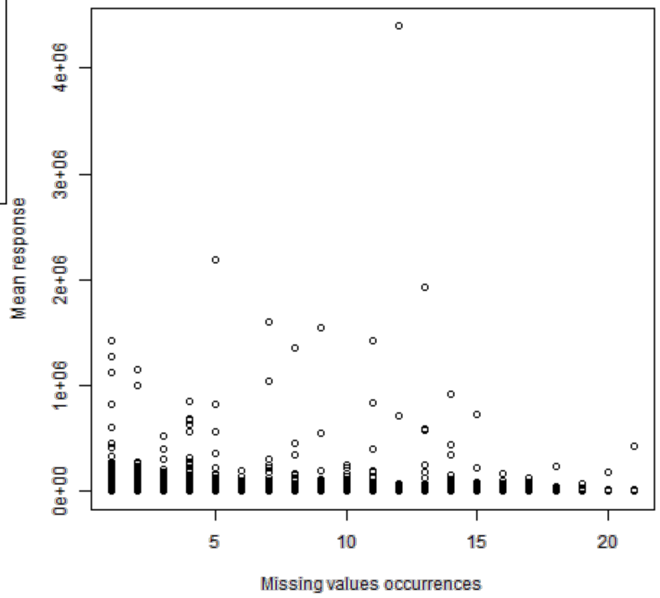

Human placental tissue

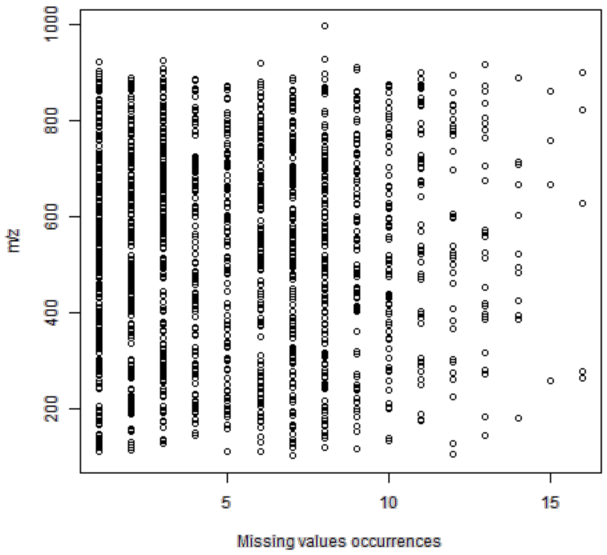

m/z

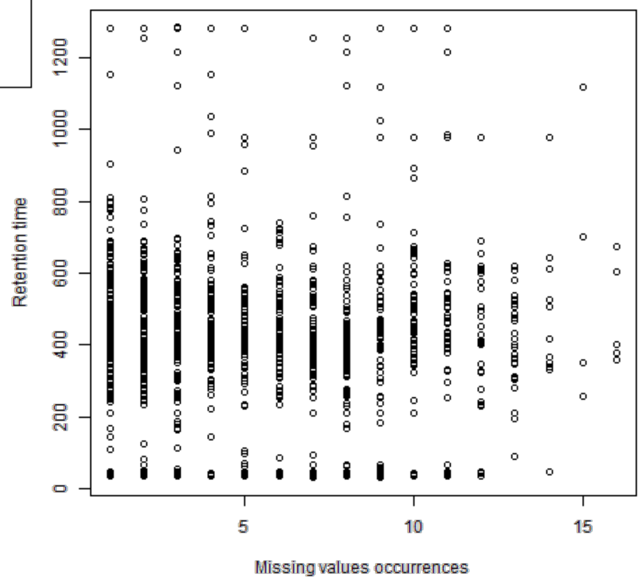

Retention time

Response

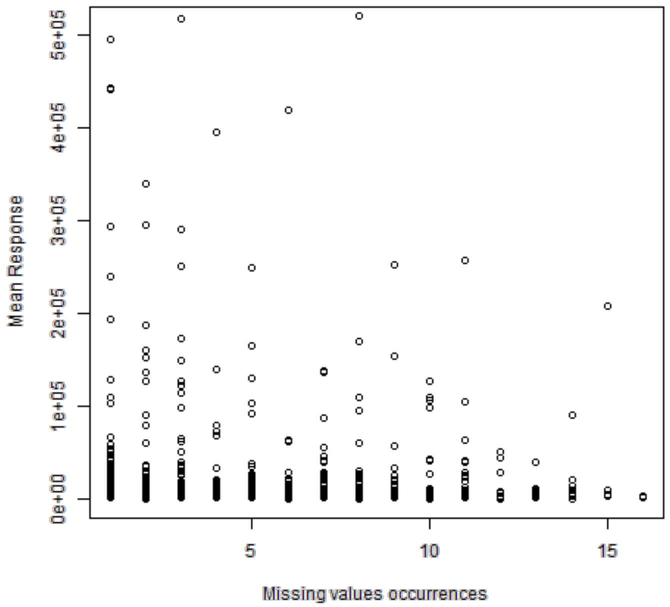

Human urine

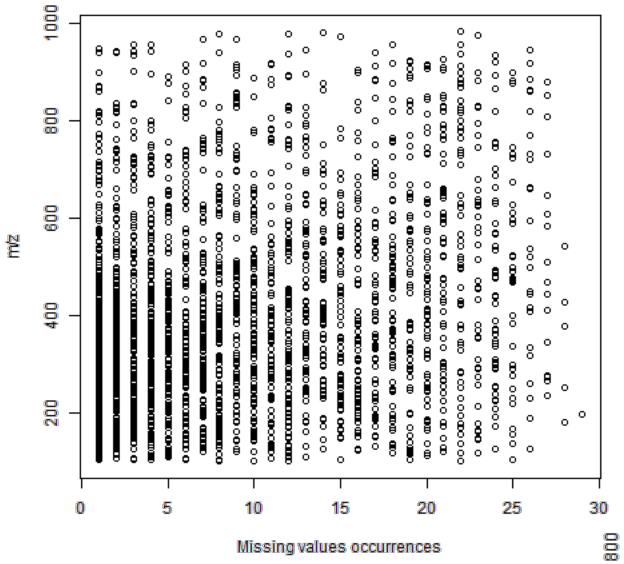

m/z

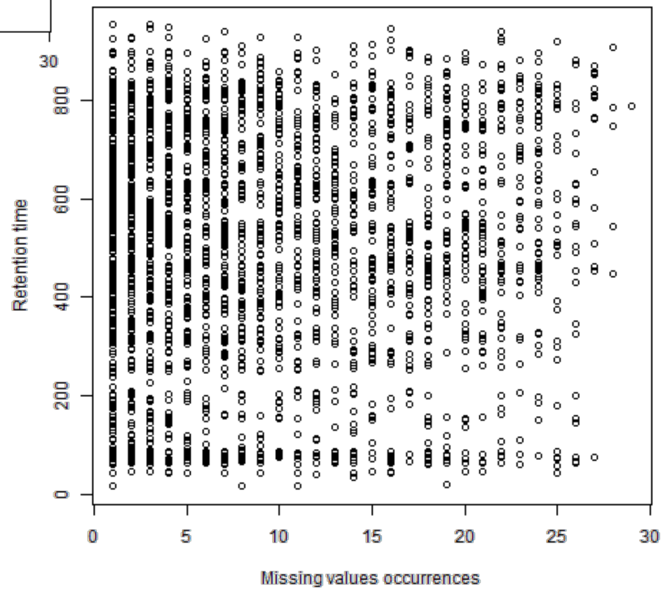

Retention time

Response

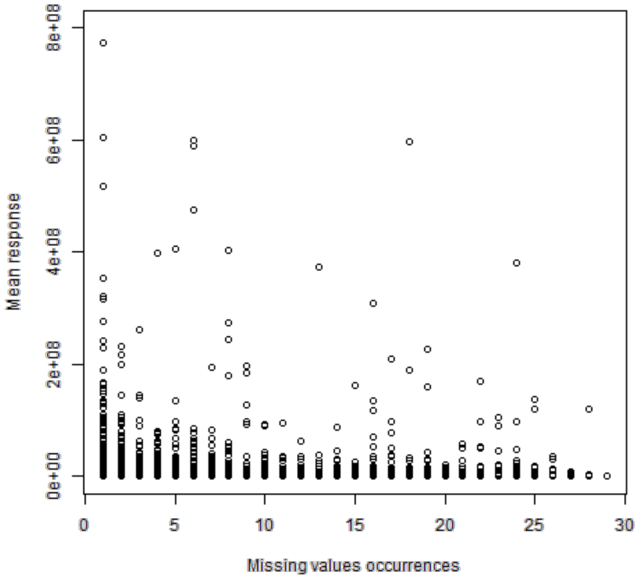

Supplement: Supplementary file 6 — Supplementary material 6 (PDF 237 kb) [file 11306_2016_1030_MOESM6_ESM.pdf]
