# Supplementary material for: Non-targeted UHPLC-MS metabolomic data processing methods: a comparative investigation of normalisation, missing value imputation, transformation and scaling
Source: Metabolomics. 2016 Apr 15;12:93. doi: 10.1007/s11306-016-1030-9 (PMC4831991; doi:10.1007/s11306-016-1030-9)
Supplement: Supplementary file 8 — Supplementary material 8 (PDF 257 kb) [file 11306_2016_1030_MOESM8_ESM.pdf]

SI 6. Percentage of metabolite features following a normal distribution before and after glog transformation. Metabolite features containing at least one class producing Shapiro-Wilk p-values<0.05 were treated as not providing a normal distribution.

**Normalisation**	**glog transformation**	**Normally distributed peaks (%)**
SUM	Not performed	39.6
	Performed	44.3
PQN	Not performed	41.6
	Performed	50.7
